# Hydrolysable tannins, physicochemical properties, and antioxidant property of wild-harvested *Terminalia ferdinandiana* (exell) fruit at different maturity stages

**DOI:** 10.3389/fnut.2022.961679

**Published:** 2022-07-29

**Authors:** Anh Dao Thi Phan, Jiale Zhang, Maral Seididamyeh, Sukirtha Srivarathan, Michael E. Netzel, Dharini Sivakumar, Yasmina Sultanbawa

**Affiliations:** ^1^ARC Industrial Transformation Training Centre for Uniquely Australian Foods, Queensland Alliance for Agriculture and Food Innovation, The University of Queensland, Indooroopilly, QLD, Australia; ^2^Department of Crop Sciences, Phytochemical Food Network Research Group, Tshwane University of Technology, Pretoria, South Africa

**Keywords:** Kakadu plum, indigenous fruit, undomesticated harvest, phytochemicals, fruit developmental stages, antioxidant properties

## Abstract

*Terminalia ferdinandiana* Exell., also known as Kakadu plum, is a wild-harvested native Australian fruit with limited information on how maturity is affecting the phytonutritional properties and bioactivities of the fruit. Thus, this study investigated changes in hydrolysable tannins, phenolic acids, sugar profile, standard physicochemical parameters, and antioxidant-scavenging capacity of wild-harvested Kakadu plum fruits at four different maturity stages, from immature to fully mature. Fruits harvested <25, 25–50, 50–75, and 75–100% degree of fullness were classified as highly immature (stage 1), immature (stage 2), semi-mature (stage 3), and fully mature (stage 4), respectively. Results showed that chebulagic acid, geraniin, chebulinic acid, castalagin, punicalagin, and gallic acid continuously decreased during fruit maturity, while elaeocarpusin, helioscopin B, corilagin, 3,4,6-tri-*O*-galloyl-*S*-glucose, and ellagic acid increased at the beginning of fruit growth (from stage 1 to 2), but decreased when the fruits reached their full maturity (stage 4). The levels of hydrolysable tannins and phenolic acids in fully mature fruits (stage 4) were significantly (*p* ≤ 0.05) lower than that in their immature counterparts (stages 1 and 2). Total phenolic content (TPC) and DPPH antioxidant radical-scavenging activity did not vary significantly between different maturity stages. Pearson's correlation coefficient test indicated that TPC and DPPH positively (*p* ≤ 0.05) correlate with most of the studied tannin compounds. Sugars (glucose, fructose, and sucrose), total soluble solid content, and titratable acidity increased during the fruit development. Furthermore, principal component analysis (PCA) revealed the difference between the immature and mature samples, based on their nutritional profile and bioactive compounds. The PCA results also suggested a considerable variability between the individual trees, highlighting the challenges of wild-harvest practice.

## Introduction

*Terminalia ferdinandiana* Exell., an endemic Australian plant, belongs to the *Terminalia* genus, which includes 250 species (1). It is a member of the Combretaceae family. In Western Australia, Northern Territory, and northern Queensland, it is commonly known as Kakadu plum, billy goat plum, and gubinge (2). Kakadu plum is a small- to moderate-sized semi-deciduous tree with smooth-skinned, fleshy ovoid drupes, a short beak, and yellow-green-colored fruits (3). The fruit of this plant has been widely used as traditional food or folk medicine by the Australian Indigenous communities (4). Since the Kakadu plum fruit possesses anti-inflammatory, antimicrobial, antioxidant, and anticancer properties (5–7), this has led to increased scientific interest in the characterization of phytochemicals, biofunctional properties, and applications. Due to its functional properties, the fruit has grown in popularity in a number of markets, including functional food ingredients, health, cosmetics, and nutraceuticals (2, 8).

Hydrolysable tannins are polyphenolic compounds with a high molecular weight, a complex molecular structure, and a relatively strong polarity (9). *Terminalia* species is well-known as a rich source of hydrolysable tannins, of which ellagitannins and gallotannins are dominant and mainly contribute to the reported health-related benefits of this plant species (10, 11). Despite the lack of commercial reference materials and the complexity of their molecular structures, studies on characterization of hydrolysable tannin compounds in Kakadu plum and elucidation of their molecular structures are limited (11). In most of the recent studies, hydrolysable tannins in Kakadu plum are estimated indirectly through the semi-quantification of their corresponding hydrolysed metabolites. A key step in quantification of ellagitannins is based on the release of hexahydroxy-diphenic acid, which undergoes spontaneous lactonization to ellagic acid under acidic condition at high temperatures (12–14). The hydrolysable tannin and phenolic acid compounds, such as ellagic acid and corilagin, were identified and quantified as predominant constituents in Kakadu plum fruit powder using high-resolution accurate mass (HRAM) spectrometry (15). Williams et al. (13) quantified the hydrolysable tannin compounds in Kakadu plum fruit as total ellagic acid equivalent and reported that the fruit possesses a higher level of ellagic acid (259.1 mg/100 g DW) than that of other common ellagic acid-rich fruits such as strawberries (4.8 mg/100 g DW) and boysenberries (5.5 mg/100 g DW). Furthermore, the total phenolic content (TPC) of Kakadu plum fruit has been reported as 6-fold higher than that of blueberry, a benchmark antioxidant-rich fruit (5, 13).

Kakadu plums are traditionally wild-harvested, and as a result, a large fluctuation in the bioactive compounds (vitamin C and ellagic acid) has been reported in Kakadu plum fruits collected from three seasons and four different maturity stages (14). Phan et al. (14) reported a positive correlation between vitamin C and an increase in fruit maturity in wild-harvested Kakadu plum. However, ellagic acid content decreased with fruit maturation. However, little is known about the impact of fruit maturation on hydrolysable tannin compounds, the accumulation of soluble sugars, and antioxidant activity. In addition, there is insufficient information about the effects of wild-harvest conditions on the variation of bioactive compounds and bioactivities. Konzack et al. (16) reported a significant variation in the levels of bioactive compounds in Kakadu plum fruits harvested from different accessions, locations, and different individual trees at the same location. Additionally, other potential factors, including changes in climatic conditions (e.g., rainfall level, solar exposure intensity, and air temperature), can affect the fruit morphology and the biosynthesis of bioactive compounds during fruit growth (14). Therefore, understanding the effect of wild-harvest conditions on the fruit quality at harvest is crucial for researchers, the Australian Indigenous community, Indigenous enterprises, and the Australian bush food industry.

**Figure 1:**
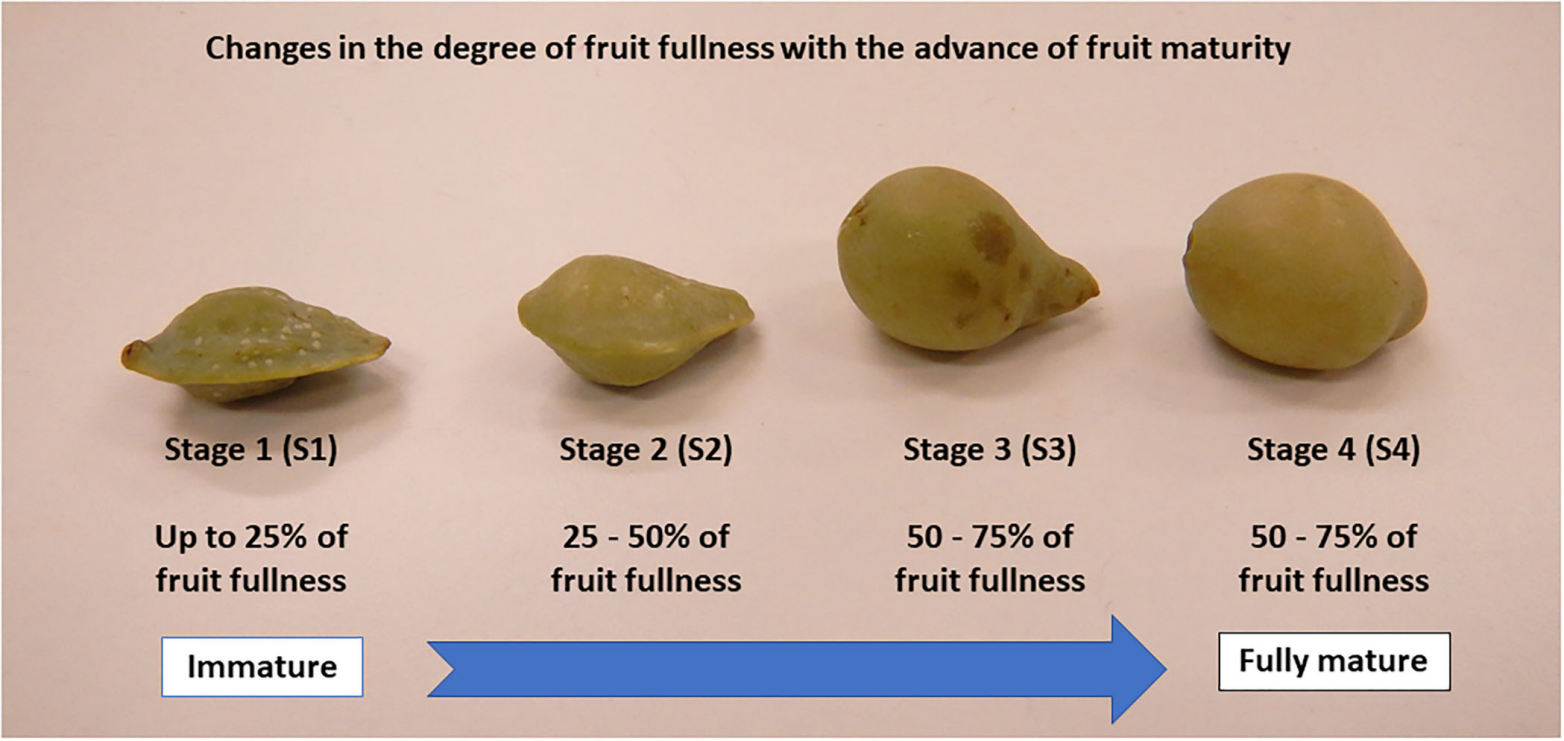
Changes in the degree of fruit fullness with an increase in fruit maturity.

In view of the above, this study investigated the effects of fruit maturity on the hydrolysable tannins and phenolic acids, physicochemical properties, sugar components, and antioxidant-scavenging activity of wild-harvested Kakadu plum fruits at four different maturity stages as described in Figure 1.

## Materials and methods

### Chemical reagents

Polyphenol and sugar standards (HPLC grade, ≥95% purity), including gallic acid, ellagic acid, corilagin, 3,4,6-tri-*O*-galloyl-*S*-glucose, castalagin, punicalagin, fructose, glucose, and sucrose, were purchased from Sigma-Aldrich (Castle Hill, NSW, Australia). All the other chemicals and solvents (HPLC/analytical grade) used throughout the study were supplied by Merck (Bayswater, VIC, Australia) or Sigma-Aldrich.

### Plant materials

Kakadu plum fruits are traditionally harvested from plants that are grown in the “wild” habitat without any human intervention, cultivation, and controlled growing environment. In this study, Kakadu plum fruits were harvested randomly from six individual trees (*ca*. 5 kg/tree) in the Thamarrurr region (Darwin, Northern Territory, Australia). The required permissions were obtained from the Northern Territory Government Parks and Wildlife Commission, and the Traditional Owners. Fruits from each individual tree were sorted into four different maturity stages from immature to fully mature levels (~200 g fruit/each maturity stage), based on differences in the degree of fruit fullness between the maturity stages as reported in previous studies (8, 17). Fruits <25, 25–50, 50–75, and 75–100% fullness were assigned to samples at maturity stage 1 (S1), S2, S3, and S4, respectively (Figure 1). A total 24 observations (4 maturity stages × 6 individual trees) were performed for each variable measured in this study, which followed a completely randomized block design.

The fruit samples were transported with dry ice in cooler boxes (4°C) to the laboratory, subsequently frozen at −35°C and freeze-dried at −48 ± 2°C for 72 h (CSK Climatek, Darra, QLD, Australia). After separating the freeze-dried pulp and seeds using a laboratory blender (Waring, Australian Scientific, Sydney, NSW, Australia), the freeze-dried fruit pulp was ground into a fine powder using a Mixer Mill (MM400 Retsch, Thermo Fisher Scientific, Brisbane, QLD, Australia). The fruit powdered samples were stored at −35°C until further analysis.

### Determination of total soluble solid content and titratable acidity

Approximately 1 g of freeze-dried powder samples was mixed with Milli-Q water (1:20; *w/v*) and vortexed for 1 min. The homogeneous mixture was centrifuged at 3,900 rpm (25°C, 10 min) (Eppendorf 5180, Hamburg, Germany). The supernatant was collected for the determination of the total soluble solid content (TSS, %) using a digital refractometer (Hanna Instruments Ltd., Leighton Buzzard, UK), and measurements of pH and titratable acidity (TA, expressed as percentage of citric acid equivalent) using an automatic titration unit (Metrohm Dosimat 765, Karl Fischer, Metrohm, Herisau, Switzerland). The analysis was conducted in triplicate.

### Extraction of bioactive compounds

Hydrolysable tannins and other phenolic compounds were extracted by mixing ~0.5 g of powdered samples with aqueous methanol (80%, *v/v*) containing 0.01 N HCl according to Bobasa et al. (15), with few modifications. The mixtures were sonicated in an ultrasonication bath (Elma Schmidbauer GmbH, Ruiselede, Belgium) for 15 min, followed by 15-min shaking at room temperature (25°C) in a reciprocating mixer (RP1812, Paton Scientific, Adelaide, SA, Australia). Next, the mixtures were centrifuged at 3,900 rpm (10 min, 25°C) (Eppendorf 5180 centrifuge). After collecting the supernatant, the residues were re-extracted twice with aqueous methanol as described above. The supernatants were combined and filtered through a 0.2 μm hydrophilic PTFE syringe filter membrane into HPLC vials for subsequent hydrolysable tannin analysis. The extraction was conducted in triplicate.

### UHPLC-HRAM MS/MS analysis

Bioactive compounds in Kakadu plum fruit extract (refer to “Extraction of bioactive compounds” section) were identified and quantified using a Thermo high-resolution accurate Q Exactive mass spectrometer (Thermo Fisher Scientific Australia Pty Ltd., Melbourne, VIC, Australia), equipped with a DIONEX Ultimate 3000 UHPLC system. The instrumental method followed a previous study reported by Bobasa et al. (15), with some modifications. The compounds were separated in a Waters^®^ HSS T3 column (150 × 2.1 mm *i.d*.; 1.8 μm) maintained at 40°C, with 0.1% formic acid as mobile phase A and acetonitrile containing 0.1% formic acid as mobile phase B. The flow rate was 0.3 ml/min and gradient elution for mobile phase B was as follows: 0–1 min, 5% B; 1–8 min, 5%−20% B; 8–15 min, 20%−45% B; 15–22 min, 45–100% B; isocratic elution at 100% B for 2 min, and recondition to 5% B for 5 min before the next injection. The mass spectrometer was operated in parallel reaction monitoring in negative electrospray ionization mode at 35,000 full-width half-maximum resolution, an AGC target value of 2e5, a maximum injection time of 200 ms, and optimized normalized collision energy from 25 to 35 eV. The inclusion list of 13 interested/targeted hydrolysable tannins and phenolic acids, with detailed information about the mass features, is presented in Supplementary Table 1. A mix-standard solution (including gallic acid, ellagic acid, corilagin, 3,4,6-tri-*O*-galloyl-*S*-glucose, castalagin, and punicalagin) was prepared in MeOH and was also included in the liquid chromatography-mass spectrometry analysis to facilitate the compound identification and development of external standard calibration curves for quantification. The Thermo Trace Finder v.5.1 software (Thermo Scientific, Brisbane, QLD, Australia) was employed for data processing.

### Determination of TPC and DPPH-free radical-scavenging activity

The TPC and DPPH-free radical-scavenging activity were used to determine the antioxidant activity of Kakadu plum fruit extract (refer to “Extraction of bioactive compounds” section). For TPC, Folin–Ciocalteu assay was applied following the procedure previously reported (18). To quantify the TPC, gallic acid was used as a standard. Results are expressed as g of gallic acid equivalent per 100 g sample on a dry weight basis (g GAE/100 g DW).

Ascorbic acid standard was used to quantify the DPPH-scavenging capacity of Kakadu plum fruit extract (18). Results are expressed as g of ascorbic acid equivalent per 100 g sample on a dry weight basis (g AAE/100 g DW).

### Analysis of sugar components

Extraction and analysis of individual soluble sugars in Kakadu plum fruit samples were conducted followed the method previously reported by Hong et al. (19), with minor modifications. Briefly, about 0.5 g of samples (in triplicate) were homogenized with aqueous methanol (62%, *v/v*) using a vortex mixer, followed by incubation in a shaking water bath at 50°C for 30 min (LSB18, Grant Instruments, Amsterdam, Netherlands). After centrifuging (3,900 rpm, 10 min, 25°C), the supernatant was collected and the pellet was re-extracted twice with 62% MeOH. The supernatants were combined and filtered through 0.2 μm hydrophilic PTFE syringe filter membrane into HPLC vials. A Shimadzu Nexera X2 UHPLC system coupled with a Shimadzu MS8045 triple quadrupole mass spectrometer (Shimadzu, Kyoto, Japan) was employed for sugar analysis. Using multiple reaction monitoring in the negative mode, soluble sugars, including fructose (*m/z* 179.2 → 113.1), glucose (*m/z* 179.2 → 89.0), and sucrose (*m/z* 341.2 → 179.2), were determined. Compound separation was performed in a Waters UPLC BEH Amide column (100 × 2.1 mm *i.d*., 1.7 μm; Waters, Dublin, Ireland) maintained at 40°C, with mobile phase A (80% acetonitrile containing 0.1% NH_4_OH) and mobile phase B (0.1% NH_4_OH). The gradient program for mobile phase B at a flow rate of 0.2 ml/min was as follows: 0–1 min, 0% B; 1–7 min, 0–40% B; and recondition to the initial condition for 5 min before the next injection. A mix standard solution, including glucose, fructose, and sucrose dissolved in Milli-Q water, was prepared for the establishment of external standard calibration curves for quantification of the soluble sugars detected in the fruit extract. The concentration of sugars is expressed as g per 100 g of sample on a dry weight basis.

### Statistical analysis

Data were calculated and presented as the mean and standard error of the mean. A general linear model procedure was applied to perform the analysis of variance between the fruit samples collected at different fruit maturity stages, followed by Tukey's multiple comparison *post-hoc* tests using Minitab 17^®^ for Windows (Minitab Inc., State College, PA, USA). A *p*-value of ≤ 0.05 was used to determine significant differences. A Pearson's correlation coefficient test was also applied to test the correlation between antioxidant activity and bioactive compounds studied. A principal component analysis (PCA), including 20 measured variables with 6 replications and a full-crossed validation, was performed to visualize the variability in the dataset, using the GraphPad Prism software ver. 9.3 (GraphPad Software, San Diego, CA, USA).

## Results and discussion

### Changes in hydrolysable tannins and phenolic acids in Kakadu plum fruits with the advance of fruit maturity

**Figure 2:**
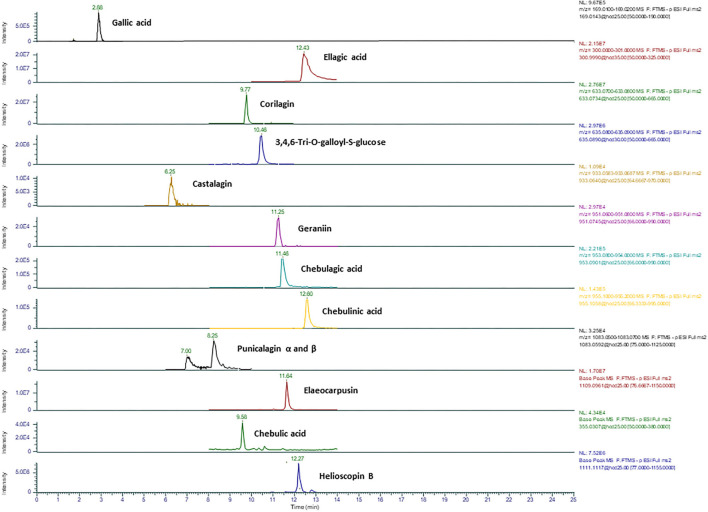
Representative ion chromatograms of 13 main hydrolysable tannins and phenolic acids detected in Kakadu plum fruit using an HRAM Orbitrap mass spectrometer.

**Figure 3:**
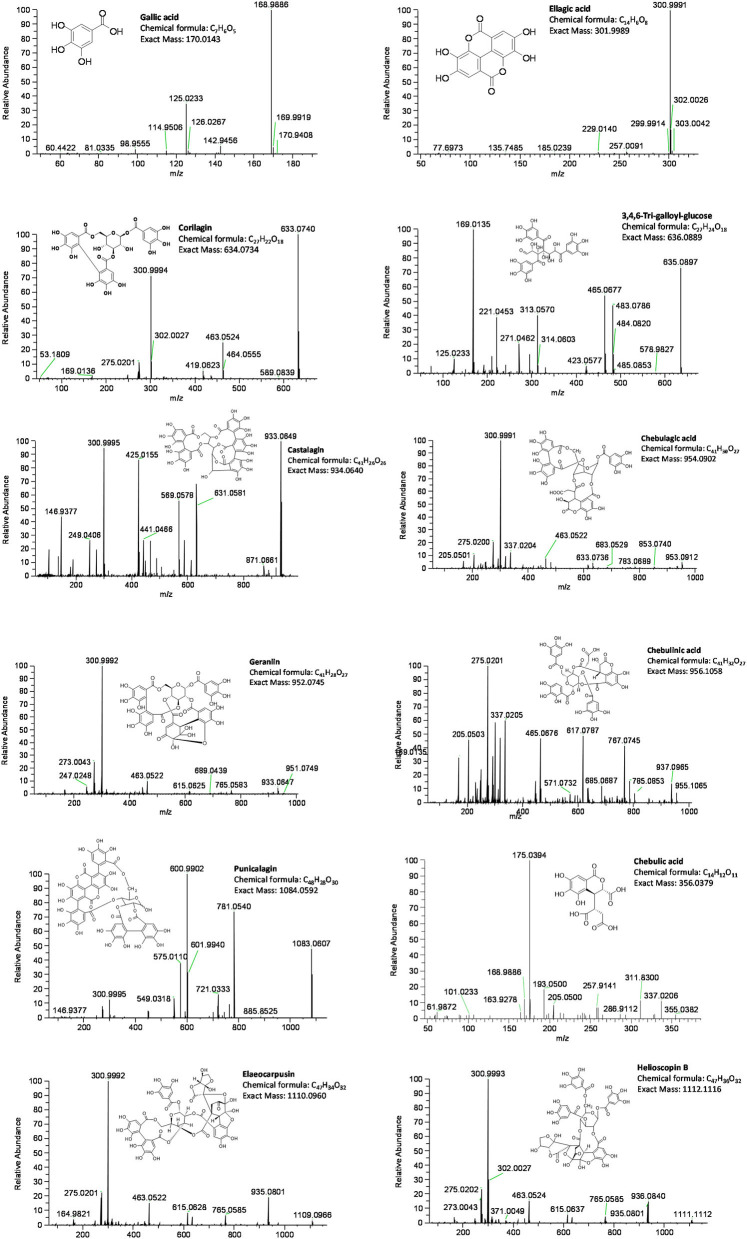
Molecular structures and mass features of the study compounds.

**Figure 4:**
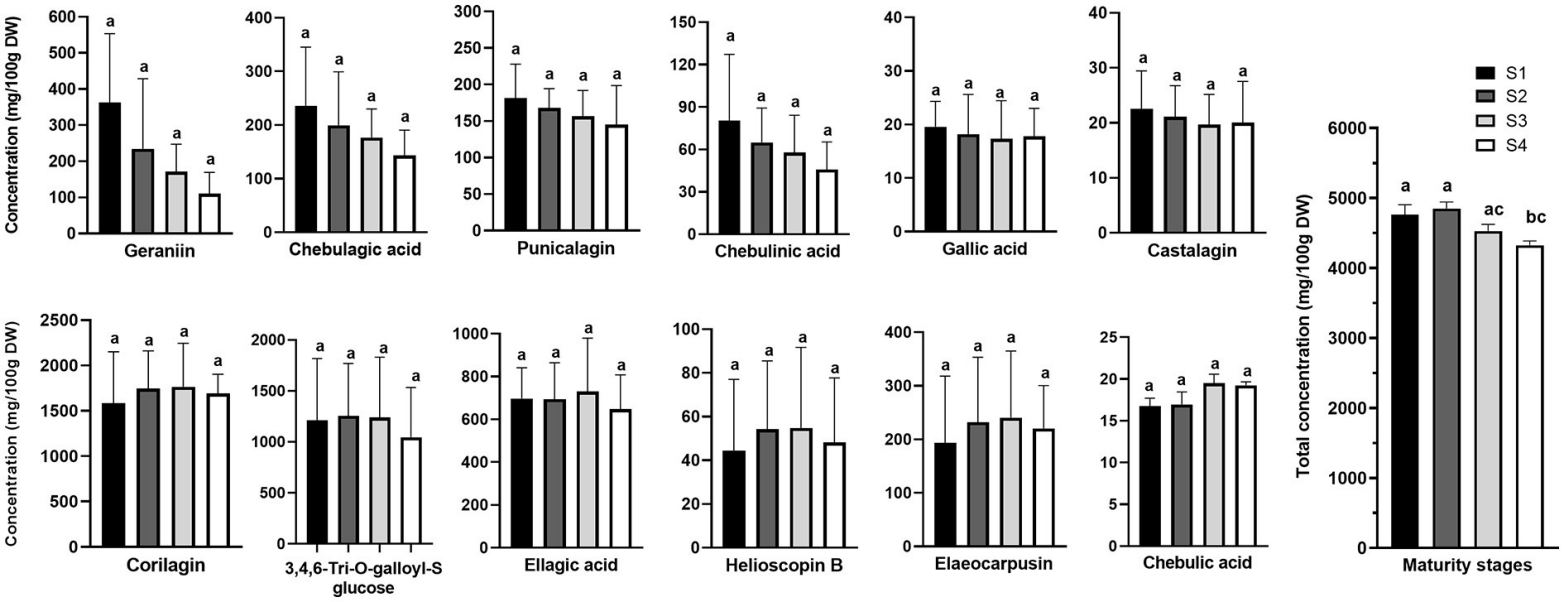
Changes in the concentrations of the study bioactive compounds in Kakadu plum fruit harvested at different maturity stages. Data presents mean ± SE, *n* = 6. S1–4 denotes different fruit maturity stages classified from immature stage (S1) to fully mature stage (S4). Different letters indicate the significant differences at *p* ≤ 0.05.

Figures 2, 3 show representative mass spectrum and MS^2^ fragmentations of 13 individual hydrolysable tannins and phenolic acids detected in Kakadu plum fruit extract. The detected and identified compounds included gallic acid, ellagic acid, corilagin, 3,4,6-tri-*O*-galloyl-*S*-glucose, castalagin, geraniin, chebulagic acid, chebulinic acid, punicalagin, and its isomer, elaeocarpusin, chebulic acid, and helioscopin B. The identification of the detected compounds was based on matching the mass features and the MS^2^ characteristic fragment ions with those of the commercial standards included in the UHLPC-HRAM-MS/MS analysis (“Chemical reagents” section) and those reported in the literature (15, 20, 21). The results of this study were consistent with previous studies, which reported the presence of the corresponding compounds in Kakadu plum fruit (3, 15, 22), as well as in other *Terminalia* species (23, 24). The results showed that corilagin has the highest concentration among the study compounds, ranging from ~1,600 to 1,800 mg/100 g DW, followed by 3,4,6-tri-*O*-galloyl-*S*-glucose (1,046–1,380 mg/100 g DW), ellagic acid (648–730 mg/100 g DW), geraniin (111–363 mg/100 g DW), elaeocarpusin (216–244 mg/100 g DW), chebulagic acid (143–246 mg/100 g DW), and punicalagin and its isomer (145–172 mg/100 g DW). All the other compounds studied were present at levels below 100 mg/100 g DW (Figure 4). This study also confirms previous findings that corilagin is one of the major tannin compounds in Kakadu plum fruit (3, 15).

Figure 4 clearly shows two opposite trends in the biosynthesis of hydrolysable tannins and phenolic acids during the fruit growth. A steady decrease was observed in the concentrations of chebulagic acid, geraniin, chebulinic acid, castalagin, gallic acid, and punicalagin with the advance of fruit maturity. In contrast, the levels of elaeocarpusin, helioscopin B, corilagin, 3,4,6-tri-*O*-galloyl-*S*-glucose, and ellagic acid were slightly increased from immature stage S1 to S3, and then decreased from S3 to the fully mature stage S4. Similarly, in pomegranate, as the fruit matures, the level of hydrolysable tannins in aril juice declines along with gallic acid and ellagic acid concentrations (25). In addition, several studies have shown a relatively higher level of hydrolysable tannins at the early maturity stage of several tannin-rich fruits such as java plum (*Syzygium cumini* Lam.), carob (*Ceratonia siliqua* L.), and different persimmon (*Diospyros kaki* Thunb.) cultivars (26–28).

Although the biosynthesis of the individual hydrolysable tannins and phenolic acids during Kakadu plum fruit development was not significantly different (*p* > 0.05), the total (sum) levels of all the study compounds at the fully mature stage (S4) were significantly lower than those measured at the immature stages (S1 and S2) (Figure 4). This suggests that hydrolysed tannins in Kakadu plum fruit decreased significantly as the fruit ripens. A decreasing trend in the total ellagic acid content (after acid hydrolysis of ellagitannins) during the ripening process of wild-harvested Kakadu plum fruits was recently reported (14, 17). The reduction in hydrolysable tannins, which is associated with the fruit maturation process, could be attributed to the polymerization of tannin compounds that can potentially bind to other macromolecules such as proteins, polysaccharides, and fibers to form the complex structures (29). According to previous research, this could have limited the extractability of tannin compounds (30). Furthermore, it has been reported that plants produce tannins as secondary metabolites to protect the plants against virus and microbe attacks during fruit development (31); for instance, the plant defense mechanism has been reported to contribute to the decrease in the content of bioactive compounds (e.g., persin, epicatechin, and catechin) with fruit maturation as reported previously in New Zealand-grown “Hass” avocado fruit (32).

### An increase in TSS content and TA during fruit development

Table 1 shows the results of TSS content, pH values, and TA of Kakadu plum fruits harvested at different stages of maturity. TSS increased from 2.0% at the immature stage (S1) to 4.1% at the mature stage (S4), whereas TA increased rapidly from 2.8% (S1) to 4.8% when the fruits were matured (S4). Furthermore, pH, which is inversely correlated with TA, showed a significant decrease (*p* ≤ 0.05) at S4. TSS and TA did not differ significantly among the maturity stages, possibly due to a large variation among individual trees that derived from the effect of wild-harvest practice. It has been reported that the quality of the fruits, grown naturally in the wild without a controlled environment, is highly variability and depends on multiple growing conditions (water availability, soil quality, temperature amplitude, nutrient availability) (16). The increase in TSS was probably derived from the accumulation of sugars during the fruit development (discussed later in the “Sugar accumulation with the advance of fruit maturity” section) as reported previously (33, 34). The higher TA content in the ripe fruit could be attributed to the accumulation of the exceptional amount of ascorbic acid in Kakadu plum fruits (up to 20% DW) at the full-maturity level (12, 14). The observed increasing trend in TA is in contrast with the decreasing trend reported for other common domestic fruits such as mango (cv. Cogshall) (35) and pomegranate (*Punica granatum* L.) (36).

**Table 1:** Total soluble solid, titratable acidity, and pH value of Kakadu plum fruit at different maturity stages.

**Maturity stage**	**TSS (%)**	**TA (%)**	**pH**
S1	2.0 ± 0.1 a	2.8 ± 0.8 a	4.03 ± 0.01 a
S2	3.3 ± 0.1 a	3.6 ± 0.6 a	3.87 ± 0.01 ab
S3	3.7 ± 0.1 a	4.5 ± 0.8 a	3.82 ± 0.01 ab
S4	4.1 ± 0.1 a	4.8 ± 0.2 a	3.80 ± 0.01 b

*Results present mean ± SE, n = 6. S1–4 denote the different maturity stages from immature (S1) to mature (S4). Different letters indicate the significant differences at p ≤ 0.05*.

### Sugar accumulation with the advance of fruit maturity

**Figure 5:**
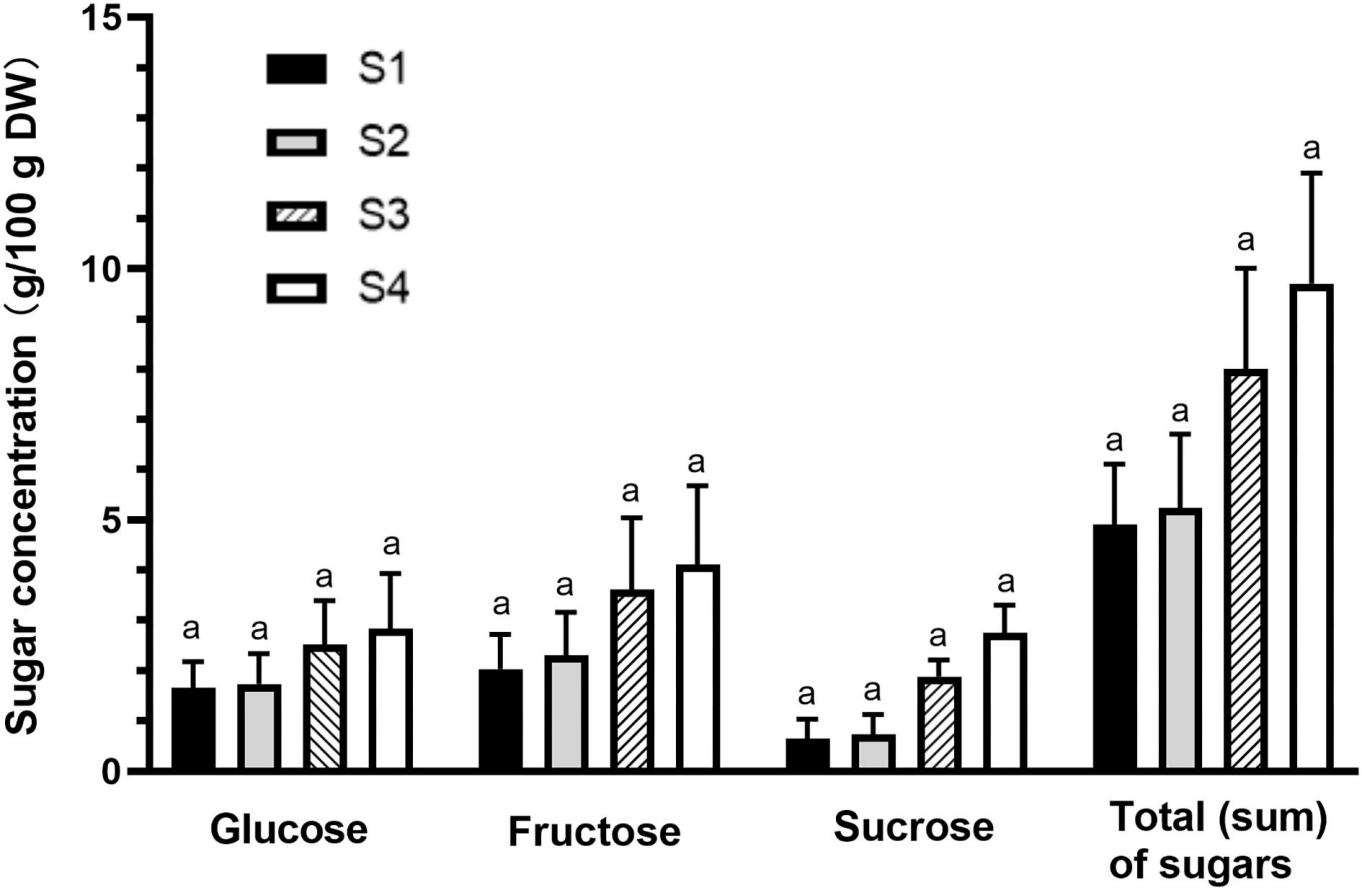
Individual sugar components and total (sum) sugars of Kakadu plum fruit harvested at different maturity stages. Results are mean ± SE, *n* = 6. S1–4 denote the different maturity stages from immature (S1) to fully mature (S4). Different letters indicate the significant differences at *p* ≤ 0.05.

The changes in the levels of main sugar components (glucose, fructose, and sucrose) and total (sum) sugar content during fruit growth are presented in Figure 5. Fructose was found as a major sugar component (2.0–4.1 g/100 g DW), followed by glucose (1.7–2.8 g/100 g DW), and sucrose (0.7–2.8 g/100 g DW). The gradual accumulation of sugars during fruit maturation led to an increase in total sugar content from 4.9 to 9.7 g/100 g DW from immature to fully mature stage, and therefore the observed increase in TSS (refer to the “An increase in TSS content and TA during fruit development” section). However, like TA and TSS results, no significant difference was observed among the four maturity stages. The low levels of glucose, fructose, and sucrose in the immature fruits could be due to their rapid usage by the cells. The early stages of fruit development require sugars to provide energy and intermediates needed for cell division and growth (37). Fruit sugars and total sugars tended to increase at late stages of development probably due to starch breakdown and continuous accumulation of sugars (37), particularly fructose, which increased the total concentration of soluble sugars, and sweetness, to a maximum level at maturity. Recently, changes in the degree of fruit fullness have been considered as maturity index for harvesting Kakadu plum fruits (17). Therefore, the obtained results on the increase of total sugar content during fruit ripening (e.g., 4.9–5.2% DW and 8–9.7% DW for the immature and semi- to fully ripened stages, respectively) may be considered a secondary fruit maturity index for Kakadu plum fruit, which together with the fruit fullness can facilitate the development of a standard harvest protocol for this wild-harvested fruit.

The sugar content observed in this study was higher than that previously reported in Kakadu plum (2.3 g/100 g DW) (16) and other *Terminalia* species, including *Terminalia citrina* [2.6 g/100 g DW; (38)] and *Terminalia chebula* [2.3 g/100 g DW; (39)], which could be due to the effects of wild-harvest practice depending on multiple environmental factors (14). Individual fruits may exhibit significant variations in their external and internal qualities depending on their location in a tree canopy (40). The differences observed between the same species could be attributed due to the effect of location, sunshine hours, and photosynthetically active radiation (40). As with this study, a large variation in total sugar content has been reported among the individual Kakadu plum trees that were harvested at the same location in Northern Territory, Australia (16). The synthesis of sugar in stone fruit during fruit development has been reported to be influenced by both agronomical and environmental factors (41), and these factors are not controlled in the wild-harvested plants like Kakadu plum as it is happening in domesticated crops. This might be explained by the variation of sugar content between the individual trees observed in this study.

### Changes in antioxidant capacity during the fruit growth

**Figure 6:**
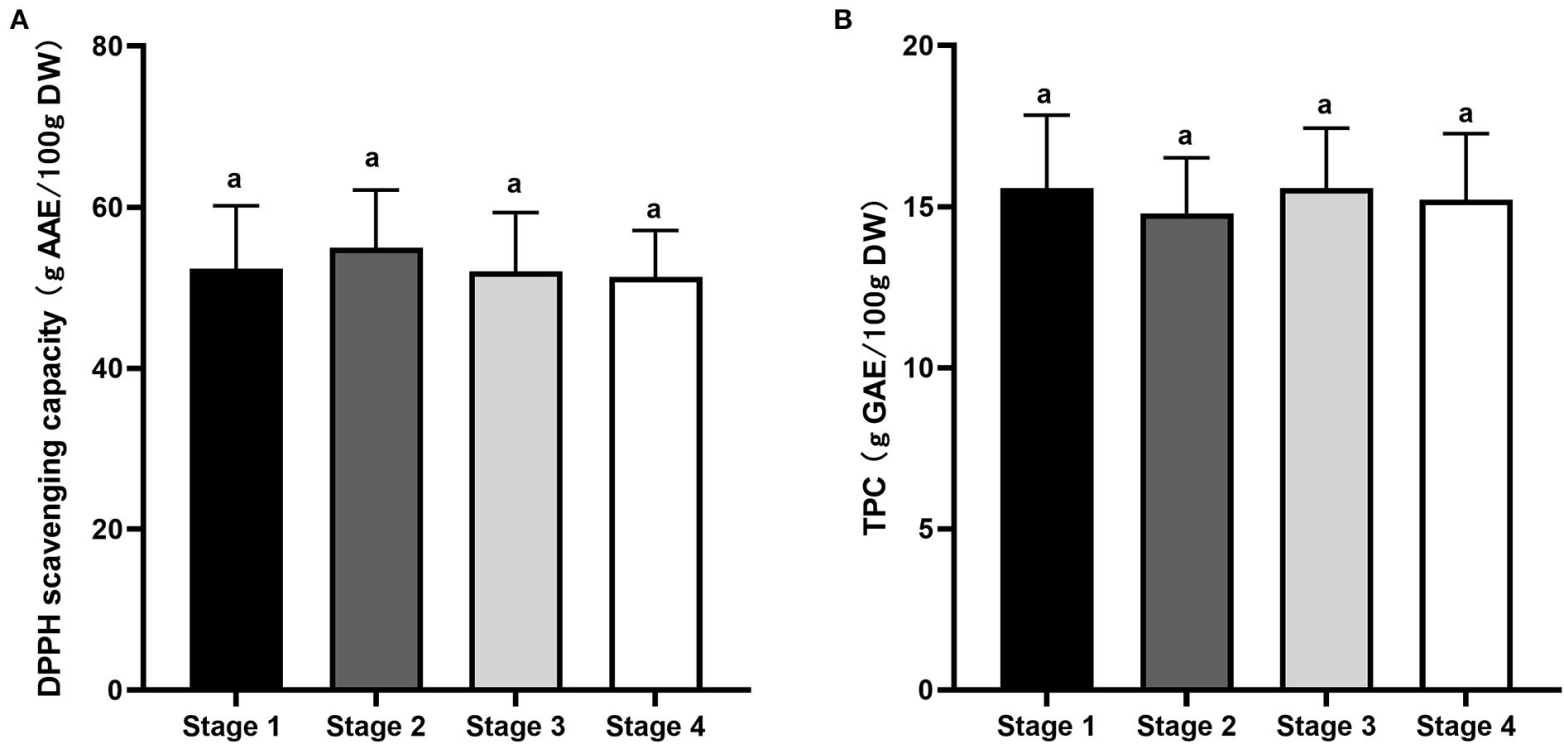
**(A)** DPPH-free radical-scavenging capacity and **(B)** total phenolic content of Kakadu plum fruit at different maturity stages. Data are mean ± SE (*n* = 6). DPPH-scavenging activity and TPC are expressed as ascorbic acid equivalent (g AAE/100 g DW) and gallic acid equivalent per 100 g dry sample (g GAE/100 g DW), respectively. Different letters indicate the significant differences at *p* ≤ 0.05.

Figure 6 shows DPPH-free radical-scavenging activity and TPC of Kakadu plum fruit harvested at different maturity stages. DPPH-scavenging activity varied from 51.4 to 55 g AAE/100 g DW during maturation, with S2 exhibiting slightly higher DPPH scavenging activity than the other maturity stages, although no statistical difference (*p* > 0.05; Figure 6A). Similarly, there was no significant (*p* > 0.05) difference in TPC values among the studied maturity stages, which was about 15 g GAE/100 g DW (Figure 6B). The obtained results are in the range of the reported TPC values of 45 accessions of Kakadu plum fruits varying from 12.2 to 50.5 g GAE/100 g DW (16). The results suggested that fruit maturity is unlikely to affect the antioxidant capacity of wild-harvested Kakadu plum fruits as the biosynthesis of plant secondary metabolites such as phenolics is considerably dependent on numerous environmental factors, including sunshine hours, soil condition, temperature, air quality, and water availability (42–45).

A Pearson's correlation coefficient test was conducted (Supplementary Table 2) to obtain a better understanding on the relationship between antioxidant capacity and the main bioactive compounds of Kakadu plum fruits. A positive correlation (*r* = 0.65, *p* <0.01) between DPPH-scavenging capacity and TPC was observed. In addition, TPC positively correlated with most of the studied hydrolysable tannin compounds, including 3,4,6-tri-*O*-galloyl-*S*-glucose (*r* = 0.55, *p* <0.01), chebulinic acid (*r* = 0.51, *p* <0.05), elaeocarpusin (*r* = 0.62, *p* <0.01), and helioscopin B (*r* = 0.55, *p* <0.01); except for castalagin (*r* = −0.41, *p* <0.05) and punicalagin (*r* = −0.39, *p* > 0.05), which were present in the samples at relatively low amounts (Figure 4). Furthermore, there was a positive correlation between DPPH-free scavenging capacity and chebulinic acid, elaeocarpusin, and helioscopin B (*r* = 0.44, 0.51, and 0.45, respectively; *p* <0.01). The results suggested that phenolic compounds, including hydrolysable tannins, could mainly contribute to the antioxidant capacity of Kakadu plum fruit, which is consistent with previous research in Kakadu plum (16), as well as other *Terminalia* species (15, 18, 46).

### Principal component analysis indicates the effect of fruit maturity and highlights the challenges of wild-harvest practice

**Figure 7:**
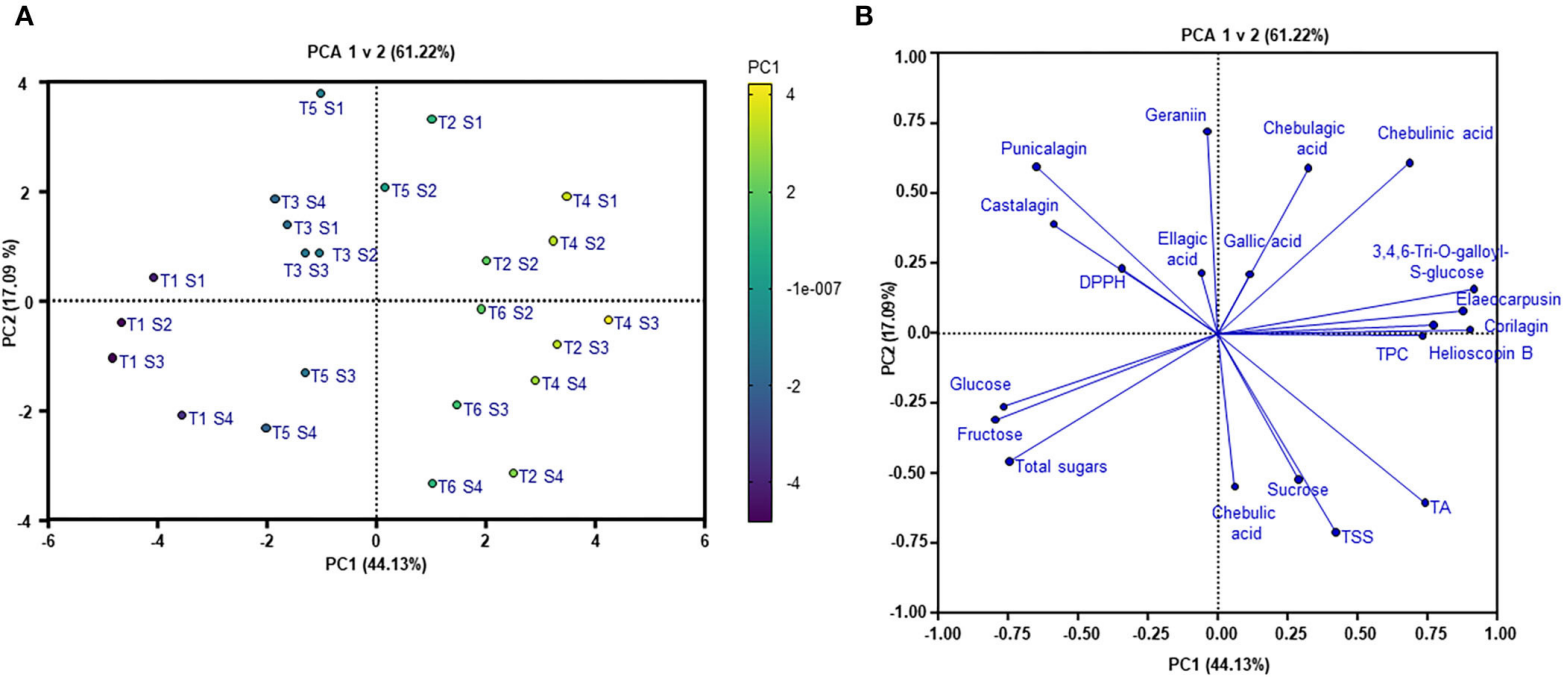
**(A)** PCA scores plot and **(B)** loadings plot. T1–T6, trees; S1–S4, maturity stages; TPC, total phenolic content; DPPH, antioxidant capacity; TA, titratable acidity; TSS, total soluble solid content.

Principal component analysis was applied to visualize the effects of fruit maturity on the changes of bioactive compounds, sugars, antioxidant capacity, and other fruit quality parameters. Generally, the PCA scores plot (Figure 7A), explaining 61.22% of the total variability (PC1 44.13% and PC2 17.09%) in the dataset, demonstrates a clear separation between the individual studied trees along the PC1. As can be seen in Figure 7A, trees 1, 3, and 5 were grouped together, while trees 2, 4, and 6 clustered in another group. The PC2, however, divides the samples into two distinguished groups according to fruit maturity levels, including the immature group (S1 and S2) and the mature one (S3 and S4). The PCA results highlighted the higher variability in the dataset influenced by variations among the studied trees. This confirms the considerable effect of wild-harvest conditions on the nutritional profile and antioxidant capacity of Kakadu plum fruit.

The PCA loadings plot (Figure 7B) reveals the extent of the contribution of measured variables to the variations observed in the dataset. In PC1, hydrolysable tannin compounds, which are found at high concentrations such as helioscopin B (*r* = 0.9), 3,4,6-tri-*O*-galloyl-*S*-glucose (*r* = 0.92), elaeocarpusin (*r* = 0.88), chebulinic acid (*r* = 0.69), and corilagin (*r* = 0.77), together with TPC (*r* = 0.73), glucose (*r* = −0.77), fructose (*r* = −0.8), and TA (*r* = 0.74), contributed to the separation among the trees. In contrast, punicalagin (*r* = 0.6), chebulinic acid (*r* = 0.61), chebulic acid (*r* = 0.55), geraniin (*r* = 0.72), total sugars (*r* = −0.46), sucrose (*r* = −0.52), and TSS (*r* = −0.710) were mainly responsible for the differences between the fruit maturity stages observed along the PC2. Furthermore, the PCA loadings plot showed that hydrolysable tannins and antioxidant capacity positively correlated with the immature fruit samples (S1, S2), whereas sugar components, TA, and TSS were positively correlated with the mature fruits (S3, S4). The obtained results highlight an opposite trend in the results that as the fruit matures more sugars and acids are accumulated, and more hydrolysable tannins are reduced.

## Conclusion

The obtained results provide a better understanding of the relationship between the fruit maturity and the accumulation of different bioactive compounds and their associated bioactivities. This can help the Australian Indigenous enterprises, growers, and scientists, as well as the food and other industries, select the appropriate maturity stages for harvesting Kakadu plum fruit for further development of functional food ingredients, pharmaceutical, and/or nutraceutical products. Generally, the results showed that fruit maturity plays an important role in determining the quality of Kakadu plum fruit at harvest. TSS, TA, and sugars increased during the fruit development from highly immature (<25% degree of fruit fullness) to fully mature stages (75–100% degree of fruit fullness), whereas phenolic compounds, including major hydrolysable tannins, exhibited a decreasing trend in the result as fruit ripens. A positive correlation between antioxidant capacity and hydrolysable tannins was observed, suggesting that tannins might mainly contribute to the bioactivity of Kakadu plum fruit. The PCA results enabled us to differentiate between the immature fruits having <50% degree of fruit fullness and the semi- to fully mature fruit samples reaching 50–100% degree of fruit fullness. Additionally, the PCA highlighted the considerable influence of wild-harvest conditions. Further studies using a larger sample size from different locations and harvest seasons/years are required to substantiate the current results.

## Data availability statement

The original contributions presented in the study are included in the article/Supplementary material, further inquiries can be directed to the corresponding author/s.

## Author contributions

AP, MN, DS, and YS: conceptualization. AP, JZ, MS, SS, and DS: methodology. AP and JZ: software. AP, MN, DS, and YS: validation. AP, JZ, MS, and SS: formal analysis. AP and JZ: data curation. AP: writing—original draft preparation. JZ, MS, SS, MN, DS, and YS: review and editing. DS and YS: supervision. YS: funding acquisition. All authors have read and agreed to the published version of the manuscript.

## Funding

Funding support from the CRC for Developing Northern Australia Limited Project AT.2.1718031 – Improving the efficiency of Kakadu plum value chains to grow a robust and sustainable Industry and the Australian Government through the Australian Research Council's (ARC) Industrial Transformation Training Centre (ITTC) for Uniquely Australian Foods (Grant number: IC180100045) is acknowledged.

## Conflict of interest

The authors declare that the research was conducted in the absence of any commercial or financial relationships that could be construed as a potential conflict of interest.

## Publisher's note

All claims expressed in this article are solely those of the authors and do not necessarily represent those of their affiliated organizations, or those of the publisher, the editors and the reviewers. Any product that may be evaluated in this article, or claim that may be made by its manufacturer, is not guaranteed or endorsed by the publisher.
