# Supplementary material for: Hydrolysable tannins, physicochemical properties, and antioxidant property of wild-harvested Terminalia ferdinandiana (exell) fruit at different maturity stages
Source: Front Nutr. 2022 Jul 29;9:961679. doi: 10.3389/fnut.2022.961679 (PMC9372433; doi:10.3389/fnut.2022.961679)
Supplement: Supplementary file 1 [file Table_1.pdf]

Supplementary Materials

Table S1. Mass spectrometric data operated in negative mode of targeted hydrolysable tannins and phenolic acids present in Kakadu plum fruit extract at different maturity stages.

Compounds	Molecular formula	Retention time (min)	$[M-H]^-$	Collision energy (eV)	Target transition ion for quantification	Transition ions for confirmation
Gallic acid	$C_7H_6O_5$	2.88	169.0142	25	168.9886	125.0233
Castalagin	$C_{41}H_{22}O_{18}$	6.25	933.0639	25	933.0649	631.0581, 425.0155<br>300.9995
Punicalagin and its isomer	$C_{48}H_{28}O_{30}$	7.0<br>8.25	1083.0592	25	600.9902	1083.0607<br>781.0540<br>300.9995
Chebulic acid	$C_{14}H_{12}O_{11}$	9.58	355.0306	25	175.0394	355.0382, 168.9886
Corilagin	$C_{27}H_{22}O_{18}$	9.77	633.0733	25	633.0740	463.0524, 300.9994
3,4,6-Tri-*O*-galloyl-*S*-glucose	$C_{27}H_{24}O_{18}$	10.46	635.0889	30	635.0897	483.0786, 465.0677<br>169.0135
Geraniin	$C_{41}H_{28}O_{27}$	11.25	951.0745	25	300.9992	933.0647, 463.0522
Chebulagic acid	$C_{41}H_{30}O_{27}$	11.46	953.0901	25	300.9991	953.0912, 463.0522<br>275.0200
Elaeocarpusin	$C_{47}H_{34}O_{32}$	11.64	1109.0960	25	300.9992	1109.0966<br>935.0801<br>463.0522
Helioscopin B	$C_{47}H_{36}O_{32}$	12.27	1111.1116	25	300.9992	1111.1112<br>463.0524<br>275.0202
Chebulinic acid	$C_{41}H_{32}O_{27}$	12.60	955.1058	25	275.0201	955.1065, 785.0853<br>465.0676
Ellagic acid	$C_{14}H_6O_8$	12.43	300.9989	35	300.9992	257.0091
