# Supplementary material for: Hydrolysable tannins, physicochemical properties, and antioxidant property of wild-harvested Terminalia ferdinandiana (exell) fruit at different maturity stages
Source: Front Nutr. 2022 Jul 29;9:961679. doi: 10.3389/fnut.2022.961679 (PMC9372433; doi:10.3389/fnut.2022.961679)
Supplement: Supplementary file 2 [file Table_2.pdf]

Supplementary Materials

Table S2. Pearson’s correlation coefficients between ellagitannins and the values of TPC/DPPH of the wild-harvested Kakadu plum fruits.

	Castalagin	Corilagin	3,4,6-Tri-O-galloyl-S-glucose	Punicalagin	Gallic acid	Ellagic acid	Chebulagic acid	Chebulinic acid	Elaeocarpusin	Chebulic acid	Helioscopin B	Geraniin	TPC	DPPH
Castalagin	1.000													
Corilagin	-0.610**	1.000												
3,4,6-Tri-O-galloyl-S-glucose	-0.506*	0.836**	1.000											
Punicalagin	0.691**	-0.410	-0.531**	1.000										
Gallic acid	0.162	0.044	0.037	0.183	1.000									
Ellagic acid	0.324	-0.192	-0.174	0.358	0.226	1.000								
Chebulagic acid	-0.279	0.594**	0.461*	0.190	-0.019	-0.035	1.000							
Chebulinic acid	-0.246	0.557**	0.748**	-0.084	0.120	0.084	0.624**	1.000						
Elaeocarpusin	-0.359	0.752**	0.822**	-0.461*	0.303	0.104	0.293	0.680**	1.000					
Chebulic acid	-0.620**	0.281	0.132	-0.338	-0.498*	-0.319	0.027	-0.109	-0.140	1.000				
Helioscopin B	-0.509*	0.738**	0.892**	-0.601**	0.190	-0.043	0.168	0.633**	0.874**	0.038	1.000			
Geraniin	-0.022	0.199	0.137	0.449*	-0.145	-0.045	0.882**	0.475*	-0.118	-0.021	-0.175	1.000		
TPC	-0.414*	0.391	0.554**	-0.389	0.120	0.188	0.229	0.506*	0.616**	-0.034	0.554**	-0.015	1.000	
DPPH	-0.201	0.338	0.380	-0.240	0.200	0.347	0.142	0.441*	0.505*	-0.048	0.451*	-0.040	0.653**	1.000
